# Towards cancer-aware life-history modelling

**DOI:** 10.1098/rstb.2014.0234

**Published:** 2015-07-19

**Authors:** Hanna Kokko, Michael E. Hochberg

**Affiliations:** 1 Wissenschaftskolleg zu Berlin, Institute for Advanced Study, Wallotstrasse 19, Berlin 14193, Germany; 2 Institute of Evolutionary Biology and Environmental Studies, University of Zurich, Winterthurerstrasse 190, Zurich 8057, Switzerland; 3 Institut des Sciences de l'Evolution, Université Montpellier, UMR5554 du CNRS, Montpellier 34095, France; 4 Santa Fe Institute, 1399 Hyde Park Road, Santa Fe, NM 87501, USA

**Keywords:** Peto′s paradox, cancer, life history, body size, sexual conflict, coevolution

## Abstract

Studies of body size evolution, and life-history theory in general, are conducted without taking into account cancer as a factor that can end an organism's reproductive lifespan. This reflects a tacit assumption that predation, parasitism and starvation are of overriding importance in the wild. We argue here that even if deaths directly attributable to cancer are a rarity in studies of natural populations, it remains incorrect to infer that cancer has not been of importance in shaping observed life histories. We present first steps towards a cancer-aware life-history theory, by quantifying the decrease in the length of the expected reproductively active lifespan that follows from an attempt to grow larger than conspecific competitors. If all else is equal, a larger organism is more likely to develop cancer, but, importantly, many factors are unlikely to be equal. Variations in extrinsic mortality as well as in the pace of life—larger organisms are often near the slow end of the fast–slow life-history continuum—can make realized cancer incidences more equal across species than what would be observed in the absence of adaptive responses to cancer risk (alleviating the so-called Peto's paradox). We also discuss reasons why patterns across species can differ from within-species predictions. Even if natural selection diminishes cancer susceptibility differences between species, within-species differences can remain. In many sexually dimorphic cases, we predict males to be more cancer-prone than females, forming an understudied component of sexual conflict.

## 1. Introduction

Animal body size is a key life-history trait in terms of the ecological niche and the associated evolutionary process [1,2]. Body size affects the ability to survive and reproduce via a diversity of mechanisms including competition for limiting resources and for mating opportunities, and predation avoidance [3,4]. Life-history theory also has to explain ‘what keeps organisms small’ (despite often documentable benefits of being larger), with studies indicating the roles of natural enemies and disease [5,6], and reviews focusing on costs of large size such as delayed reproduction and high energy requirements during or after growth [7,8]. Here we focus on an underappreciated cost: because growing to a larger body size requires more cell divisions (this is a simple corollary of the fact that animals vary much more in cell number than cell size), it is difficult to build a larger body without elevating the cancer risk experienced by the organism.

Most of what is known about the biology of cancer risk is based on experimental cell cultures, laboratory mice and correlative evidence found in human populations. Evolutionary ecologists rarely include cancer in their lists of sources of mortality for non-domesticated animals, which reflects a tacit assumption that parasites, predation and starvation are of overriding importance in the wild. We argue that this is an oversimplification because cancer, even if rarely directly observable as a cause of death, can have significant evolutionary implications [9]. This is for two reasons. First, whether or not a species is cancer-prone, field methods are unlikely to reveal much mortality directly attributable to cancer—as long as we make the sensible assumption that suboptimal physical performance of an animal (due to an incipient tumour or any other disease) will make it an easier target for predators or parasites. Consequently, for organisms living in the wild, cancer-related deaths would typically be attributed to other, more direct causes, long before a tumour is visible (the spectacular contagious tumours of Tasmanian devils being an obvious exception). A second line of evidence that cancer risk is being moulded by natural selection comes from the so-called Peto's paradox. This paradox is the lack of a statistically significant association between cancer risk and body size or longevity across species [10–12], despite reasoning based on first principles (assuming constant risk per cell division) predicting that a positive association should exist [13].

The details of the prediction that larger, longer-lived organisms should, all else being equal, be more vulnerable to cancers are based on two interrelated phenomena: standing cells as ‘targets’ for mutagenesis (viruses, UV, etc.) and the vulnerability of cell replication to random mutation [14,15]. In an intraspecific context, there is good evidence that this vulnerability is heightened for entities—either tissues or whole organisms—that are larger than their peers. In the context of different tissues, Tomasetti & Vogelstein [16] recently showed that the total number of healthy stem cell replications over a lifetime in humans explained a significant amount of variation in incidence among 31 cancer types. The authors suggest that random mutations occurring at cell division is a mechanism that could explain this effect (but see [15,17]). In a similar vein, at the whole organism level, height predicts the risk of some cancers, especially bone cancers, in humans [16–21] (but see [22]). There is also evidence for higher cancer incidence in larger dog breeds and giant laboratory mice [21,23].

These results projected onto interspecific patterns suggest that evolution towards larger body sizes will increase cancer vulnerability, unless some lifestyle, physiological or cellular mechanism evolves that limits increases in cancer risk [10,11]. One possible mechanism that influences risk is that larger species tend to have a slower pace of life (they occur at the slow end of the so-called fast–slow continuum, e.g. [24,25]; see [26] for a cellular-level view). However, the association between large bodies and slow lives is a double-edged sword. If cells replicate at a slower rate, the organism will on the one hand experience less oncogenetic risk per time unit, but on the other hand, a slow-paced organism has to maintain its body for a longer amount of absolute time to achieve any reproductive success. As a whole, therefore, it remains the case that the larger number of cell divisions required to build a large body creates cancer risks that larger-bodied organisms need to cope with. Peto's paradox suggests that solutions have been found [11], and the same message arises in a recent study among tissues in humans: Noble *et al*.'s reanalysis [15] of the Tomasetti & Vogelstein dataset [16] reveals that cancer incidence saturates with the total number of stem cell divisions. This is suggestive of a role of natural selection either in limiting the size or stem cell replications in the most cancer-prone tissues and/or in lowering the probability of obtaining cancer through specific mechanisms in these same tissues.

Given the diversity and complex interlocking of different life-history traits, and their ecological and evolutionary interactions with carcinogenesis, deciphering simple causal pathways is likely to be challenging. Here we employ a simple mathematical argument to investigate how we should expect body size to relate to cancer risk in different environmental settings. First, in §2, we consider how different empirical representations of cancer ‘risk’ can be encapsulated into a mathematical function. With this in hand, we then develop and analyse mathematical models to address two questions: does cancer risk constrain body size evolution (§3), and conversely, when do we expect selection for improved cancer defences (§4)? Finally, in §5, we compare our model and results with previous theoretical studies and in §6 discuss limitations and future directions.

## 2. Towards ‘cancer-aware’ life-history modelling

Absolute cancer risk (hereafter ‘cancer risk’) is the probability that a person contracts the disease over a fixed period of time, usually for a range of specific ages or over a lifetime. Cancer risk is generally calculated from epidemiological data, but how could it be mechanistically modelled?

There are various suggestions in the literature [27] as well as recent empirical evidence [16] that, in humans, cancer risk scales straightforwardly with the total number of cell divisions. A linear fit to the full dataset in Tomasetti & Vogelstein [16] gives: 10^11^ divisions → approximately 10^–1^ lifetime risk; 10^7^ divisions → 10^–4^ lifetime risk. On a log–log scale one could derive a simple prediction that if increasing body size by a factor of 10 requires 10 times as many cell divisions (an assumption that we shall discuss in §5), then each magnitude of body size increase should lead to 10^3/4^ = 5.6-fold cancer risk. It also follows that body sizes beyond approximately 10^12^ stem cells should not exist in nature, as cancer risk would then reach 100%. (A human being is estimated to contain 3.72 × 10^13^ cells [28], but not all of these are obviously stem cells.) However, incidences in Tomasetti and Vogelstein's dataset begin to saturate in tissues beyond approximately 10^9^ total cell divisions, suggesting either that natural selection has resulted in these tissues being differentially protected from cancer, and/or constrained in size and/or other mortalities occur before cancer, meaning lower effective cancer incidence [15]. Noble *et al.* [15] additionally showed that when considering families of tissues, their slopes were approximately unity, further indicating that natural selection can explain the overall pattern.

The above human-centred discussion would create the wrong predictions in an interspecific comparison, for example, by ignoring all selective processes that may have occurred to make interspecific cancer incidences more equal than they would be based on cell counts alone. A perhaps more important shortcoming is that ‘risk’ without reference to lifespan does not make much sense in an across-species context. In other words, while it makes sense to quantify a lifetime risk for a human population where each member has roughly the same expected lifespan (in the absence of any specific cancer), an attempt to encapsulate the idea of risk with a single number across organisms with markedly different lifespans would lead to a biased view of the problem. Consider a hypothetical cancer that an organism cannot avoid, in the sense that its incidence is almost 100% by the age of 2 years. If the organism is a free-living mouse, it may well have reproduced and also died of other causes by this time; if it is an elephant, it would not be anywhere near maturity by the time its life was cut short by cancer. For this reason, we derive, below, explicit cumulative probabilities for cancer that is specified for each age, *t*, of the animal, rather than a single number for risk.

We also consider the mortality rate that is associated with causes of death other than cancer (extrinsic mortality, denoted *μ*). Empirical data indicate that age-specific patterns show very wide variation [29] and thus we simply assume a constant mortality rate over time. If the extrinsic mortality rate is *μ*, then the expected lifespan (in the absence of cancer) is *L* = 1/*μ*.

A life-history model will also have to assume a schedule of reproduction. In nature, there is an astounding diversity of such scheduling [29], from age-related increases in reproductive success to declines. To avoid biasing our attention towards extreme patterns (that might only apply in specific taxa), we take the middle ground position: we assume that reproductive success neither increases nor decreases with age; instead we assume a constant rate of reproductive success that accumulates throughout life as long as the individual is cancer-free. In organisms that live for several years, this would imply that each additional year of life is assumed to add equally much reproductive success as any of the earlier ones, but we also intend our model to apply to shorter lived organisms (where lifespans are mere fractions of years). Our model, therefore, uses continuous time, where lifespan calculations are equally valid whether lifespan falls below or remains above the value of one unit of time.

Our steps towards a ‘cancer-aware’ life-history model use a simplified version of eqn (2) in [30] (see [10,31] for its original derivations)
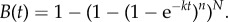


**Figure 1.**
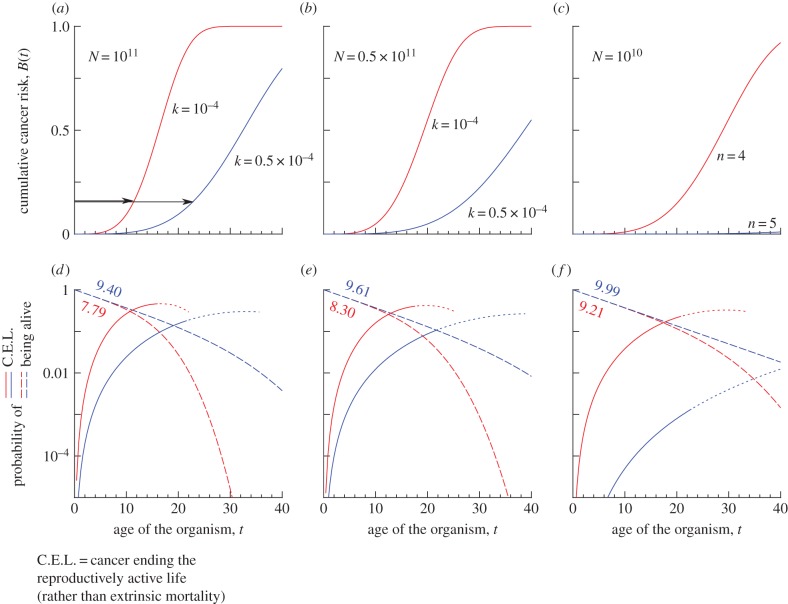
(*a*–*c*) *B*(*t*), the cumulative probability (conditional on the individual being alive) that cancer has occurred in at least one of the *N* cell lineages, as a function of age *t*, when *n* = 4 and *k* = 10^–4^ unless indicated differently in the figure. In each example, the blue curve has better cancer defences than the red curve, either because (*a,b*) its *k* is lower, or (*c*) because there is an additional defence mechanism that increases the number of rate-limiting steps from *n* = 4 to *n* = 5. (*d*–*f*) The life-history consequences of (*a*–*c*), assuming a constant extrinsic mortality rate of *μ* = 0.1: the proportion of individuals alive (dashed lines) would decrease linearly in a log-scale plot if there was no cancer; the downcurving from linearity indicates the effects of cancer. The numbers give the expected lifespan, which would be *L* = 10 in the complete absence of cancer. Solid curves give the probability, for each age *t*, that cancer is the cause whenever a reproductive career ends at that *t*. Dotted line style is used when fewer than 1% of individuals are alive from that *t* onwards, and we do not plot the curve beyond fewer than 0.1% being alive; this helps to emphasize that most individuals end their lives during a stage where cancer's role is increasing, but the overall incidence may remain low throughout in some cases, e.g. in (*e*) where we assume a small body size *N*.

The quantity *B*(*t*) (examples plotted in figure 1*a*–*c*) gives the probability that an organism has cancer by time *t* (where *t* = 0 is the time point of fertilization). Here, *k* is the rate of a single lineage undergoing one rate-limiting step towards the multi-stage process of carcinogenesis, expressed e.g. in steps occurring per year. We assume that the organism ends its reproductive career if *n* rate-limiting steps have happened in any of its *N* cell lineages—i.e. it has cancer. We assume that while the cancer may not have ended its life yet, realized reproductive success is zero from this time onwards. We also assume that cancers occurring during the growth (rather than maintenance) phase of an organism can be neglected; hence *N* is assumed constant (see §5).

We interpret *k* to combine the rate of a cell dividing with the probability of the rate-limiting step (mutation leading to cancer) happening. For example, Calabrese & Shibata [27] give the per-division estimate 10^–6^ for a specific gene target, and one division every 4 days; this would give a rough estimate of 365/4 × 10^–6^ ≈ 10^–4^ steps per year for our *k* (note that our notation differs from theirs, as our model structure is somewhat different; in §5 we shall also consider how the growth phase and the maintenance phase of an organism may complicate the issue).

We have 1 − e^−*kt*^ as the probability that a single rate-limiting step has already happened by time *t*, and 1−(1 − e^−*kt*^)*^n^* the probability that not all steps have happened yet in the same focal cell lineage. Note that *k* will be lower for organisms with a slow ‘pace of life’ (few cell divisions per unit time), but an organism might also be able to reduce *k* without reductions in cell division rate if it has better defences against cancer, e.g. mechanisms to recognize and kill tumour cells, or ‘smart’ morphological arrangements such as that found in the gut epithelium (see discussion in [14]: because of being the site of digestive fluid production, cells here have to be continually replaced and the risk created by the unavoidably fast cell replication rate appears to be minimized by morphology that mechanistically forces cells to flow unidirectionally from physically separate crypts into the gut via villi—meaning that any abnormalities cannot easily spread into neighbouring crypts before they are simply digested away).

The above equation as a whole takes the probability that all *N* lineages are healthy so far, (1−(1 − e^−*kt*^)*^n^*), and forms its complement to yield the probability *B*(*t*), of cancer having already emerged in at least one cell lineage, as indicated above.

Figure 1*a*–*c* shows examples of the behaviour of *B*(*t*) over time. Reducing *k* by 50% doubles the time (see arrows in figure 1*a*) it takes for cumulative cancer risk to reach any pre-specified value; reducing *k* by 75% would quadruple the time (since 1/(1–0.75) = 4), and so on. In absolute time, the delay is longer if the organism has already reached sufficiently old age for cancer to be a significant risk (the arrows are longer the nearer they are drawn to the upper end of the curves in figure 1*a*). Consequently, at young age, the curves depicting the proportion of individuals still alive (dashed lines in figure 1*d*–*f*) are nearly linear on a log scale: here extrinsic mortality (which we assume to operate age-independently) removes far more individuals than cancer from the pool of reproductively active individuals. The more the curves deviate downwards from linearity, the more individuals have been removed due to cancer. The numbers associating with these curves in figure 1*d*–*f* indicate expected lifespan, with all causes of death combined. These numbers are obtained by integrating *A*(*t*)(1–*B*(*t*)), where *A*(*t*) is the probability that the organism has avoided death by extrinsic mortality (*A*(*t*) = e^–*μt*^) for *t* time units (in figure 1, we assume *μ* = 0.1, such that average lifespan would reach *L* = 10 if cancer was not an issue). Figure 1*d*–*f* also gives the age-dependent probability that cancer (rather than extrinsic mortality) is the cause of a reproductive career ending at time *t*.

It is useful to comment on the differences between the different examples in figure 1. In figure 1(*a*,*d*), we assume a rather large organism that could in principle live (on average) 10 years. Cancer does significantly reduce its lifespan, and better mechanisms to delay its onset (the difference between the red and the blue curves) would lead to a strong improvement of lifespan from 7.8 to 9.4 years (figure 1*d*). However, if such mechanisms are not available, a smaller but still decidedly non-negligible increase—from the original 7.8 in figure 1*a* to 8.3 years in figure 1*b*—can be achieved if the organism simply does not grow as large. One interesting interpretation is that the different curves could relate to different sexes in sexually size dimorphic species. If females are smaller (figure 1*b*) than males (figure 1*a*), then they might escape cancer more often even if the defences against cancer were equally strong (same-coloured curves in figure 1).

The effect of body size potentially dramatically changing the role of cancer in life-history evolution is most visible in figure 1*c*. Here we have changed *n* (rather than *k*) to reflect a potentially very efficient way to delay cancer: here an organism has evolved to increase the number of rate-limiting steps that need to occur before cancer can occur (this additional control mechanism itself needs to be ‘broken’ before cancer can occur; see [10,11] for discussion and evidence). While cancer is now very efficiently delayed (figure 1*c*), we here also simultaneously assume that the organism is one magnitude smaller than the individuals of figure 1*a*. For this reason, cancer remains a limited problem (lifespan 9.21 was reached) even if the additional control is not in place.

To proceed beyond isolated examples, we now ask two questions:

(1) Does cancer risk constrain body size evolution? For an organism with parameters *μ*, *k*, *n* and *N*, how costly (in terms of the proportion of expected reproductively active lifespan lost) is it to have a specified, say 10%, increase in body size? We assume that ‘all else is equal’, that is (i) the organism attempts to increase *N* without reducing *k* via a slower pace of life or better cancer defences, and (ii) extrinsic mortality *μ* remains constant. Note that within a species, extrinsic mortality can either increase with body size, e.g. because increasing growth rate beyond an optimum makes individuals more vulnerable to food shortages [7], or decrease with body size, should the individual grow beyond the size range of some of its potential predators. Across species, evidence points to slower scheduling of life (longer lifespans and generation times) with increasing body size [32], suggesting that a decrease is the general pattern.
(2) When do we expect selection for improved cancer defences? For an organism with parameters *μ*, *k*, *n* and *N*, how large is the potential benefit of improved cancer defences in terms of increased reproductively active lifespan? In other words, if *k* is reduced by, say, 10%, or *n* is increased, how much longer will the organism live?

**Figure 2.**
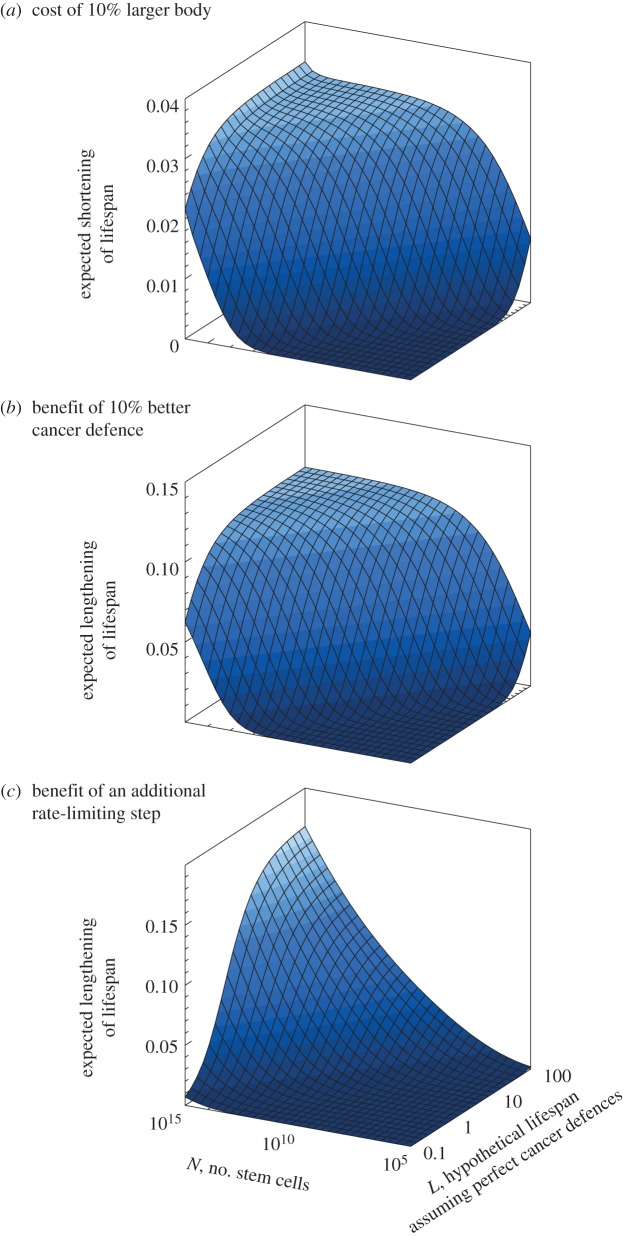
(*a*) Cost, expressed as the proportion of lifespan lost, of growing 10% larger than the baseline; (*b*) benefit, expressed as the proportional increase in expected lifespan, of reducing *k* (the rate at which each cell lineage undergoes rate-limiting steps towards cancer) by 10%; (*c*) benefit, again expressed as the proportional increase in expected lifespan, of increasing *n* by one step (to *n* = 4). Baseline parameters are *k* = 10^–4^, *n* = 3, *μ* = 1/*L* with *L* as indicated on the *y*-axis, and *N* as indicated on the *x*-axis. To interpret values, e.g. *L* = 10 combined with a cost of 0.02 means that such an organism will shorten its expected lifespan from 10 years to (1–0.02) × 10 = 9.8 years—all else being equal losing an expected 2% of its reproductive success—if it grows 10% larger from its baseline of *N* cells.

Figure 2 exemplifies the answers using *n* = 3 and *k* = 10^–4^ (alternative values give generally similar shapes). We will consider each of the two questions in turn.

## 3. Does cancer risk constrain body size evolution?

In figure 1, we used relatively large differences in body size to form our examples: interpreting a change from figure 1*b* to 1*a* as a 100% increase in body size, the corresponding changes in expected lifespan are 8.30 → 7.79 (a 6% decline) or 9.61 → 9.40 (a 2% decline). This illustrates two insights (i) there is clearly not a universal, fixed ratio describing how dangerous in terms of cancer risk it is to grow larger; but (ii) these risks can be significant: a several per cent reduction in expected lifespan is not trivial.

In figure 2*a*, which investigates a much larger parameter range than figure 1, we explore the generality of these insights. To make the comparisons relevant for micro-evolutionary change and/or intraspecific differences (e.g. males and females of a size dimorphic species), we investigate how a more modest change in body size (10% larger) translates to a shorter expected lifespan, but do this for a much larger range of body size values than in figure 1; we also vary extrinsic mortality widely to yield different values of *L* = 1/*μ*, which is the expected lifespan an organism would reach if defences against cancer were perfect.

To interpret figure 2*a*, it is important to understand where a small-bodied versus a large-bodied organism is likely to be located with respect to the two horizontal axes *L* and *N*. The axis reflecting the number of cells, *N*, is clear: it takes more cells (and cell divisions) to grow to a large size than to a small size, and as such figure 2*a* shows that large organisms, for a given *k*, *n* and *μ* (the ‘all else is equal’ assumption), will risk a much larger proportional reduction in expected reproductively active lifespan than will small organisms. With the numerical values of figure 2*a*, the reduction (the cancer risk cost) is, maximally, 3.3% of lifespan lost for a 10% increase of body size.

How easy is it for an individual to benefit from increased body size despite the cost? The benefits of being large are obviously strongly system dependent, and they can also depend on the individual's sex [33]. Assuming, as we do, that reproductive success accrues throughout cancer-free life, then an individual who lives a 3.3% shorter life should enjoy increased reproductive success of 1/(1–0.033) = 1.035-fold, per time unit, compared to its smaller peers; if this magnitude (or higher) of a reproductive benefit exists, then the heightened cancer risk will not prevent net selection from favouring larger individuals. We consider these percentages to represent biologically achievable values, given that larger individuals may have better access to food and other resources, including (for males) access to females. In species with a polygynous mating system where body size is a significant predictor of outcompeting conspecifics, rewards of being somewhat larger than conspecifics could be much larger than a few per cent. It follows that individuals, especially males in sexually dimorphic species, may evolve in a direction that increases the incidence of cancer.

On the other hand, the result also means that body size evolution can be associated with a substantial cost of reduced lifespan due to cancer (despite the fact that most causes of death may remain non-cancer related). If there are other costs of being large [7], then cancer risk may well tip the balance from further increases being favoured to being counter-selected.

In reality, ‘all else is not equal’ with respect to at least two parameters: *μ* and *k*. The effect of extrinsic mortality *μ* is visible along the *L*-axis (*L* = 1/*μ* gives the expected lifespan were the individual able to avoid all cancers). Given that lifespans generally increase with body size [32,34], large-bodied organisms are generally at the high end of both the *N-* and *L*-axes. This is precisely the region where the cancer-risk costs of increasing body size are significant. In other words, if an individual is already large and long-lived (slow life history), becoming larger still is much costlier than a similar percentage increase at the fast end (small and short lived) of the life-history spectrum.

The effect of *k* is not directly visible in figure 2*a*, but repeating the calculation for a lower value of *k* leads to a figure with smaller costs (not shown). This is logical since reducing *k* by a specific factor will delay the onset of cancer by the same factor (see [30], where the notation is somewhat different—a specific factor *d* is used to highlight this effect).

How realistic are the above figures? For most organisms, we simply lack the data; for humans, we know more, where e.g. for females, a 10 cm increase in height has been estimated to yield a relative risk of 1.14 for all cancers combined [21]. Assuming that the body mass index (mass/height^2^) remains unchanged while height increases from 1.65 to 1.75 m, a 10 cm height difference translates into a body size difference of 12.5%. The 14% increase in cancer risk for a 12.5% increase in body size may appear to represent a somewhat steeper relationship than what we have been predicting in figure 2, but it is important to note that an increase in risk and the proportion of lifespan lost are not directly comparable. This is because the latter calculation also includes other sources of mortality. Thus, a 14% higher risk (at each age) will, in reality, impact lifespan by less than 14%, because other sources of mortality remain unchanged and will mask some of cancer's potential effects.

## 4. When do we expect selection for improved cancer defences?

In contrast to the considerations in §3 where *k* and *n* were kept fixed, cancer defence itself may be an evolving quantity. Figure 2*b* depicts the benefits of reducing *k* by a specified factor (here exemplified with a 10% reduction), and figure 2*c* shows the benefits of increasing *n* (in this example from *n* = 3 to *n* = 4). Comparing figure 2*b*–*c* to figure 2*a* reveals that increased benefits occur at the same parameter values where increasing body size is costly in terms of cancer risk. This is intuitively clear: those situations where further increases of body size lead to a severe penalty in terms of cancer risk are also those where reductions in cancer risk can pay off.

In the light of Peto's paradox, the interpretation of figure 2*b*–*c* is somewhat different, however, from figure 2*a*. For simplicity, we go through our argument using reductions in *k* as the cancer defence, but similar arguments apply for increases in *n*. Large and hence ‘slow’ organisms are located where *N* and *L* are both large. Modest (10%) reductions in *k* will here lead to very significant (greater than 10%) increases in expected reproductively active lifespan, but the same reduction in *k* can also yield no benefit when *N* or *L* are small. This is key to understanding why ‘all else will not be equal’: for the same initial rate *k* at which cells go through oncogenetic steps, there are life-histories that create strong selection to achieve reductions of *k*, and others where evolutionary innovations that reduce cancer risk bring about virtually no benefit. The latter category involves small-bodied organisms that cannot live long due to extrinsic mortality; thus better protection against cancer would remain invisible to selection.

The conclusion, therefore, is that large-bodied organisms are expected to harbour strong cancer defences, but only if they are also able to escape other causes of death sufficiently long such that pushing the likely age of cancer onset towards older ages has a biologically significant effect on fitness.

It is important to remember that a lower *k* can be achieved through two fundamentally different mechanisms. First, as mentioned in §2, the cell division rate itself can be lower in an organism of a larger body size. This will automatically lower *k*, which is beneficial if all else was equal, but, importantly, all else won't be equal between slow and fast lives. We do not currently have models that explicitly contrast the benefit of slow life (dangerously fast replication of cells is avoided) against the cost (the fitness gains through reproduction will now also be gained at a slower pace). In life-history theory, the magnitude of the detrimental effects will also depend on the level of extrinsic mortality and also on the type of population regulation (see [35]).

Even if the details of this argument are best left for further study (e.g. because cancer cells, once they exist, divide at a rate that deviates from those in healthy tissue), it is an intriguing idea that a sluggish ‘pace of life’ (e.g. low fecundity per unit time) could be partially an adaptation to keep cancer risk at bay in large and long-lived organisms (see also [21]). In the light of cancer biology, such organisms try to combine what appears difficult: their task is to build a large body (necessitating many cell divisions) and also maintain it for longer than other organisms. This may only be achievable if the speed of cellular-level activities relating to growth and reproduction is not excessive, as otherwise *k* would be too large to permit long lifespans. It is clearly of potential relevance for life-history models of the slow–fast axis, given that being ‘slow’ often evolves despite its costs (if extrinsic mortality is unchanged, a slow organism will achieve less reproductive success in its lifetime compared with faster conspecifics). Explicit models that assume a trade-off between *k* and reproductive success per unit time could shed light on this issue. Given lack of direct evidence this is perhaps best left an open question, but see [36] for tantalizing evidence supporting such ideas, in the context of cell growth rates in cell cultures from short- and long-lived rodents.

Second, any process that protects the organism against cancer has the effect of lowering *k*: potential mechanisms range from improved clearing of infections (many cancers are known to have infectious causes, e.g. chronic viral hepatitis B or C infection can lead to liver cancer) to more efficient identification and killing of pre-cancer cells [11]. Regardless of the mechanism, it is important that evolutionary innovations are often discrete (e.g. extra copies of tumour suppression genes), and this has consequences for the interpretation of model results (figure 2) in intra- and interspecific contexts. Consider, for example, that there is often (but not always [33]) selection for males to be larger than females. Figure 2*a* then predicts that cancer risk may be higher for males than females. Would males then also evolve stronger defences against cancer, as figure 2*b* might predict? The answer is potentially yes, but with a strong qualifier: if the innovation is of a discrete kind (e.g. 10% reduction in *k*) it may be selected for more strongly in males than in females, but unless the innovation arises in a sex-limited chromosome, it is likely to be expressed in both sexes. The entire population then undergoes a reduction in *k*, and within a species the larger cancer risk of larger bodies is still maintained after the selective sweep.

The interpretation of figure 2 is consequently different for within- and across-species comparisons. Across species, the expectation of a stronger reduction in *k* in organisms with large *N* applies. Even so, figure 2*b*–*c* only graphs the selective advantage of employing a mechanism for reducing *k* or increasing *n*, *if such a mechanism exists and is not constrained by other trade-offs*. Removing all prospects of cancer is generally difficult and/or cannot be done without compromising growth and reproduction (i.e. fitness costs); our figures assume no such cost of the acting mechanism. If no feasible mechanism to reduce *k* or to increase *n* is at hand, then the message of figure 2*a* applies at full strength: further increases of body size are constrained because the required additional cell divisions would cause too great a reduction in expected lifespan, due to cancer.

## 5. Comparison with other models, model limitations

Although our model does not yield analytical solutions, its numerical solutions are accurate—there are ways to consider the mean and the distribution of the cancer-free lifespan, and ask how many deaths are due to cancer as opposed to other causes. This potentially gives added clarity to past study based on evolutionary simulations [37]. We also show that it is straightforward to combine body size and extrinsic mortality in the same model, and that inclusion of the latter is important for understanding life-history evolution in the face of disease risk, and disease resistance for different life-history strategies.

Our model treats cell lineages similarly to [27], though we interpret our *N* as proportional to the number of cells in the entire body. Calabrese & Shibata [27] focus on one organ—the colon—as a colony of stem cells, each dividing at a constant rate over time, but the initial growth from zygote to the fixed colony size is not explicitly modelled. Their focus, however, is not on the quantities we derive, i.e. how strongly natural selection will favour or disfavour increases in body size, or reductions in the rate at which rate-limiting steps occur (i.e. cancer defence).

Nunney [10] takes a somewhat similar approach to deriving the probability of cancer, but interprets the fitness consequences in a simpler way than we do. He notes that the selection coefficient *s* for preventing a cancer that is lethal before reproductive age equals *p*, the incidence of that cancer; for cancers with later onset age, *s* < *p*. Our model differs from his in that we explicitly consider cancer prevention in terms of lifespan when cancer can realistically only be delayed, not indefinitely prevented. Thus, the fitness benefits of a delay arise, in our model, through a longer career of offspring production until death and/or cancer stop(s) this accumulation of reproductive success. This is why it becomes essential to consider other sources of mortality together with the size of the organism: the effects of these two factors appear equally strong (figure 2) and they are also expected to covary.

There is also intriguing variability between models regarding whether one focuses on the entire organism as one ‘blob’ of a large number of stem cells, or as a collection of tissues with independently evolving defences. Nunney [10] focused on the latter case (while also noting the possibility of controls that impact all tissues), and found, as expected, that as the size (number of cells) of a tissue increases, cancer protection for that tissue should also increase. It is likely to be an empirical question as to whether baseline protection within an organism should be affected by tissue specialization in protection (and vice versa). Recent work by Noble *et al.* [15] suggests that different tissue types in humans have different levels of cancer protection, but these authors could not evaluate whether this is amplified expression of a given mechanism, or additional mechanisms.

There is also the question of how the growth and maintenance phase of an organism's life should be modelled. We intentionally sidestepped this question, simply noting that one requires at least *N* – 1 cell divisions to proceed from a zygote to an adult individual consisting of *N* cells. If the initial ontological growth is rapid and then followed by a prolonged maintenance phase where stem cells divide to yield daughter cells that replace dying cells, the true number of risky divisions will be higher. The risks related to these two phases are not necessarily identical [38]. Degregori [38] presented evidence and arguments indicating that many if not most oncogenic mutations occur during ontogeny. He argues that increases in cancer incidence with age (and, therefore, the total cumulative number of stem cell divisions) is due to age-associated alternations in cells, tissues and their environments that increase selection for oncogenic events.

Our choice to ignore the problem of ontogeny versus lifetime cell production is probably conservative, in the sense that we consider the smallest possible difference in the number of cell divisions between organisms differing in size. In reality, if large organisms need to first grow large [10] and then maintain a larger number of cell divisions throughout life, then the steepness of the surface depicting costs and benefits in figure 2 is likely to increase.

## 6. Conclusion and future directions

Our modelling should be considered a first step in integrating cancer risk with other sources of mortality and the slow–fast continuum. We hope to have voiced the message that while cancer biologists should not remain unaware of evolutionary principles [12,15,30,39], evolutionary ecologists should also not remain ignorant that multicellular organisms evolve both life-history traits and cancer protection traits, given cancer risks (a problem that probably impacts all metazoan life—see [40] for tumours in *Hydra*). Importantly, Peto's paradox does not mean that all organisms experience cancer as an equally important problem. Instead, it highlights that correlations within and across species can be different, despite both reflecting the same problem: an attempt to grow large could lead to significantly higher cancer risk, but how seriously this impacts fitness will also depend in part on extrinsic mortality.

We encourage more cancer-aware life-history modelling (see also [39]). As our model is no more than a first step in this direction, future models could usefully scrutinize the process of mutation accumulation when organisms first grow and then, tissue-specifically, maintain a certain body size. It should also be remembered that organisms differ in whether they have reached their final size at (or before) maturity; little appears to be known about cancer in relation to indeterminate growth, despite the long lives of many of such organisms. Finally, mathematical models can yield insights into the temporal process of evolution: that is to what extent empirical patterns are consistent with life history traits and cancer defences co-evolving, for example in single, matched alternative steps, or rather major changes in (for example) defence followed by more continual changes in body size and/or longevity.

Another step towards a cancer-aware life-history theory is to consider in detail what is required to achieve reproduction in the context of slow and fast schedules. We have highlighted the possibility that a slow pace of life (in *any* tissue) could in principle be an adaptation to lower the rate of cell divisions. But since virtually all steps in processes that lead to reproductive success are ultimately achieved through cells dividing, the solution to life's trade-offs is clearly not to minimize divisions either. Elucidating the costs and benefits that build the relevant trade-offs could be the first steps towards a theory that explains differences in replication rate across organisms as well as across tissues, including differences between the sexes. This last point is evolutionarily very significant, given that males (by definition, sperm-producers) are a source of much of the mutational load in sexually reproducing populations [41]. Selection for rapid spermatogenesis is one mechanism that can make males evolve to ‘accept’ higher cancer risk [42]; thus sexual conflict in species with multiple matings can involve conflict over how cancer risk is managed, in addition to many other life-history features [43].

Finally, theory predicts that the force of natural selection on life-history traits such as body size and on cancer protection mechanisms will be age-specific [44,45]. Future models should explore how age-specific extrinsic mortalities (e.g. predation, disease) and cancer dynamics interact to produce both life-history and anti-cancer traits [39]. For instance, it is an empirical fact that cancer incidence increases at older ages in humans, and the interplay of the cumulative probabilities derived here and the lowered force of selection at advanced ages [46] merits further attention. It would be interesting to know if this same effect is manifested in life-history traits such as cell replacement rate (the ‘pace of life’), and whether selection on early-life performance contributes to increased incidence in cancers late in life: this would be a cancer-oriented twist on theories of ageing [45,46]. As these and other unexplored phenomena are inherently complex, they will require mathematical models to yield empirically testable predictions.

## References

[1] Werner EE Gilliam JF 1984 The ontogenetic niche and species interactions in size-structured populations. Ann. Rev. Ecol. Syst. 15, 393–425. (10.1146/annurev.es.15.110184.002141)

[2] de Roos AM Persson L 2013 Population and community ecology of ontogenetic development. Princeton, NJ: Princeton University Press.

[3] Arendt JD 1997 Adaptive intrinsic growth rates: an integration across taxa. Q. Rev. Biol. 72, 149–177. (10.1086/419764)

[4] Clutton-Brock TH Huchard E 2013 Social competition and selection in males and females. Phil. Trans. R. Soc. B 368, 20130074 (10.1098/rstb.2013.0074) 24167304 PMC3826203

[5] Hochberg ME Michalakis Y deMeeus T 1992 Parasitism as a constraint on the rate of life history evolution. J. Evol. Biol. 5, 491–504. (10.1046/j.14209101.1992.5030491.x)

[6] Michalakis Y Hochberg ME 1994 Parasitic effects on host life-history traits—a review of recent studies. Parasite 1, 291–294. (10.1051/parasite/1994014291) 9140497

[7] Blanckenhorn WU 2000 The evolution of body size: what keeps organisms small? Q. Rev. Biol. 75, 385–407. (10.1086/393620) 11125698

[8] Metcalfe NB Monaghan P 2003 Growth versus lifespan: perspectives from evolutionary ecology. Exp. Gerontol. 38, 935–940. (10.1016/S0531-5565(03)00159-1) 12954479

[9] Vittecoq M 2015 Animal behaviour and cancer. Anim. Behav. 101, 19–26. (10.1016/j.anbehav.2014.12.001)

[10] Nunney L 1999 Lineage selection and the evolution of multistage carcinogenesis. Proc. R. Soc. Lond. B 266, 493–498. (10.1098/rspb.1999.0664) PMC1689794 10189713

[11] Caulin AF Maley CC 2011 Peto's paradox: evolution's prescription for cancer prevention. Trends Ecol. Evol. 26, 175–182. (10.1016/j.tree.2011.01.002) 21296451 PMC3060950

[12] Caulin AF Graham TA Wang L-S Maley CC 2015 Solutions to Peto's paradox revealed by mathematical modelling and cross-species cancer gene analysis. Phil. Trans. R. Soc. B 370, 20140222 (10.1098/rstb.2014.0222) 26056366 PMC4581027

[13] Peto R 1977 Epidemiology, multistage models, and short-term mutagenicity tests. In The origins of human cancer, vol. 4 (eds Hiatt HH Watson JD Winsten JA), pp. 1403–1428. Cold Spring Harbor Conf. on Cell Proliferation New York, NY: Cold Spring Harbor Laboratory Press.

[14] Cairns J 1975 Mutation selection and the natural history of cancer. Nature 255, 197–200. (10.1038/255197a0) 1143315

[15] Noble R Kaltz O Hochberg ME 2015 Peto's paradox and human cancers. Phil. Trans. R. Soc. B 370, 20150104 (10.1098/rstb.2015.0104) 26056357 PMC4581036

[16] Tomasetti C Vogelstein B 2015 Variations in cancer risk among tissue can be explained by the number of stem cell divisions. Science 347, 78–81. (10.1126/science.1260825) 25554788 PMC4446723

[17] Ashford NA 2015 Cancer risk: role of environment. Science 347, 727 (10.1126/science.aaa6246) 25678650

[18] Mirabello L 2011 Height at diagnosis and birth-weight as risk factors for osteosarcoma. Cancer Causes Control 22, 899–908. (10.1007/s10552-011-9763-2) 21465145 PMC3494416

[19] Rodriguez C Calle EE Fakhrabadi-Shokoohi D Jacobs EJ Thun MJ 2002 Body mass index, height, and the risk of ovarian cancer mortality in a prospective cohort of postmenopausal women. Cancer Epidemiol. Biomarkers Prev. 11, 822–828. 12223425

[20] Gunnell D Okasha M Smith GD Oliver SE Sandhu J Holly JMP 2001 Height, leg length, and cancer risk: a systematic review. Epidemiol. Rev. 23, 313–342. (10.1093/oxfordjournals.epirev.a000809) 12192740

[21] Nunney L 2013 The real war on cancer: the evolutionary dynamics of cancer suppression. Evol. Appl. 6, 11–19. (10.1111/eva.12018) 23396311 PMC3567467

[22] Smith GD Hart C Upton M Hole D Gillis C Watt G Hawthorne V 2000 Height and risk of death among men and women: aetiological implications of associations with cardiorespiratory disease and cancer mortality. J. Epidemiol. Commun. Health 54, 97–103. (10.1136/jech.54.2.97) PMC1731616 10715741

[23] Leroi AM Koufopanou V Burt A 2003 Cancer selection. Nat. Rev. Cancer 3, 226–231. (10.1038/nrc1016) 12612657

[24] Promislow DEL Harvey PH 1990 Living fast and dying young: a comparative analysis of life-history variation among mammals. J. Zool. 220, 417–437. (10.1111/j.1469-7998.1990.tb04316.x)

[25] Okie JG 2013 Effects of allometry, productivity and lifestyle on rates and limits of body size evolution. Proc. R. Soc. B 280, 20131007 (10.1098/rspb.2013.1007) PMC3712421 23760865

[26] Savage VM Allen AP Brown JH Gillooly JF Herman AB Woodruff WH West GB 2007 Scaling of number, size, and metabolic rate of cells with body size in mammals. Proc. Natl Acad. Sci. USA 104, 4718–4723. (10.1073/pnas.0611235104) 17360590 PMC1838666

[27] Calabrese P Shibata D 2010 A simple algebraic cancer equation: calculating how cancers may arise with normal mutation rates. BMC Cancer 10, 3 (10.1186/1471-2407-10-3) 20051132 PMC2829925

[28] Bianconi E 2013 An estimation of the number of cells in the human body. Ann. Hum. Biol. 40, 463–471. (10.3109/03014460.2013.807878) 23829164

[29] Jones OR 2014 Diversity of ageing across the tree of life. Nature 505, 169–172. (10.1038/nature12789) 24317695 PMC4157354

[30] Boddy AM Kokko H Breden F Wilkinson GS Aktipis CA 2015 Cancer susceptibility and reproductive trade-offs: a model of the evolution of cancer defences. Phil. Trans. R. Soc. B 370, 20140220 (10.1098/rstb.2014.0220) 26056364 PMC4581025

[31] Nunney L 2003 The population genetics of multistage carcinogenesis. Proc. R. Soc. Lond. B 270, 1183–1191. (doi:10.1098lrspb.2003.2351) 10.1098/rspb.2003.2351 PMC1691357 12816658

[32] Evans AR 2012 The maximum rate of mammal evolution. Proc. Natl Acad. Sci. USA 109, 4187–4190. (10.1073/pnas.1120774109) 22308461 PMC3306709

[33] Fairbairn DJ Blanckenhorn WU Székely T 2008 Sex, size and gender roles. Oxford, UK: Oxford University Press.

[34] Martin AP Palumbi SR 1993 Body size, metabolic rate, generation time, and the molecular clock. Proc. Natl Acad. Sci. USA 90, 4087–4091. (10.1073/pnas.90.9.4087) 8483925 PMC46451

[35] Jeschke JM Gabriel W Kokko H 2008 r-strategists/K-strategists. In Encyclopedia of ecology (eds Jørgensen SE Fath BD), pp. 3113–3122. Oxford, UK: Elsevier.

[36] Seluanov A 2008 Distinct tumor suppressor mechanisms evolve in rodent species that differ in size and lifespan. Aging Cell 7, 813–823. (10.1111/j.1474-9726.2008.00431.x) 18778411 PMC2637185

[37] Roche B Sprouffske K Hbid H Missé D Thomas F 2013 Peto's paradox revisited: theoretical evolutionary dynamics of cancer in wild populations. Evol. Appl. 6, 109–116. (10.1111/eva.12025) 23396800 PMC3567476

[38] DeGregori J 2013 Challenging the axiom: does the occurrence of oncogenic mutations truly limit cancer development with age? Oncogene 32, 1869–1875. (10.1038/onc.2012.281) 22751134 PMC3670419

[39] Brown JS Cunningham JJ Gatenby RA 2015 The multiple facets of Peto's paradox: a life-history model for the evolution of cancer suppression. Phil. Trans. R. Soc. B 370, 20140221 (10.1098/rstb.2014.0221) 26056365 PMC4581026

[40] Domazet-Loso T Klimovich A Anokhin B Anton-Erxleben F Hamm MJ Lange C Bosch TCG 2014 Naturally occurring tumours in the basal metazoan *Hydra*. Nat. Comm. 5, 4222 (10.1038/ncomms5222) 24957317

[41] Hedrick PW 2007 Sex: differences in mutation, recombination, selection, gene flow, and genetic drift. Evolution 61, 2750–2771. (10.1111/j.1558-5646.2007.00250.x) 17976181

[42] Lewis Z Price TAR Wedell N 2008 Sperm competition, immunity, selfish genes and cancer. Cell. Mol. Sci. 65, 3241–3254. (10.1007/s00018-008-8238-4) PMC11131609 18581051

[43] Rice WR Gavrilets S (eds). 2014 The genetics and biology of sexual conflict. New York, NY: Cold Spring Harbor Laboratory Press.

[44] Hamilton WD 1966 The moulding of senescence by natural selection. J. Theor. Biol. 12, 12–45. (10.1016/0022-5193(66)90184-6) 6015424

[45] Partridge L Barton NH 1993 Optimality, mutation and the evolution of ageing. Nature 362, 305–311. (10.1038/362305a0) 8455716

[46] Sherratt TN Wilkinson DM 2009 Big questions in ecology and evolution. Oxford, UK: Oxford University Press.

